# Characterization of the MMP/TIMP Imbalance and Collagen Production Induced by IL-1β or TNF-α Release from Human Hepatic Stellate Cells

**DOI:** 10.1371/journal.pone.0153118

**Published:** 2016-04-05

**Authors:** Sacha Robert, Thomas Gicquel, Aude Bodin, Vincent Lagente, Elisabeth Boichot

**Affiliations:** UMR991 INSERM, Université de Rennes 1, Rennes, France; University of Navarra School of Medicine and Center for Applied Medical Research (CIMA), SPAIN

## Abstract

Inflammation has an important role in the development of liver fibrosis in general and the activation of hepatic stellate cells (HSCs) in particular. It is known that HSCs are themselves able to produce cytokines and chemokines, and that this production may be a key event in the initiation of fibrogenesis. However, the direct involvement of cytokines and chemokines in HSC (self-)activation remains uncertain. In this study, the effects of pro-inflammatory cytokines IL-1α and β, TNF-α, and IL-8 on the activation state of HSCs were examined, in comparison to the pro-fibrogenic mediator TGF-β1. LX-2 cells were stimulated for 24 or 48 hours with recombinant human form of the pro-inflammatory cytokines IL-1α and β, TNF-α, and IL-8, and also the pro-fibrogenic mediator TGF-β1. Two drugs were also evaluated, the anti-TNF-α monoclonal antibody infliximab and the IL-1 receptor antagonist anakinra, regarding their inhibitory effects. In LX-2 human HSC, treatment with TGF-β1 are associated with downregulation of the metalloproteinase (MMP)-1 and MMP-3, with upregulation of tissue inhibitor of metalloproteinase (TIMP)-1, collagen type I α1, collagen type IV α1, α-SMA, endothelin-1 and PDGF-BB. Cytokines and chemokines expression were found to be downregulated, excepting IL-6. In contrast, we observed that LX-2 exposure to IL-1, TNF-α and IL-8 can reverse the phenotype of pro-fibrogenic activated cells. Indeed, MMP-1, MMP-3 and MMP-9 were found elevated, associated with downregulation of α-SMA and/or PDGF-BB, and a greater expression of IL-1β, IL-6, IL-8, CXCL1 and CCL2. Lastly, we found that infliximab and anakinra successfully inhibits effects of TNF-α and IL-1 respectively in LX-2 cells. Infliximab and anakinra may be of value in preclinical trials in chronic liver disease. Overall, our results suggest that (i) pro-inflammatory mediators exert complex effects in HSCs via an MMP/TIMP imbalance, and (ii) targeting IL-1 signaling may be a potentially valuable therapeutic strategy in chronic liver diseases.

## Introduction

Fibrosis is a common pathologic consequence of a wide variety of chronic liver diseases, including hepatitis B and C virus infections, alcoholic liver disease and nonalcoholic fatty liver disease/nonalcoholic steatohepatitis (NAFLD/NASH), and results from an accumulation of extracellular matrix (ECM) following the activation and proliferation of hepatic stellate cells (HSCs). In fact, fibrosis is a pivotal pathological process in the progression to severe cirrhosis and the loss of liver function [1]. HSCs and portal fibroblasts are considered to be the primary sources of ECM during fibrogenesis [2]. However, activated HSCs can also contribute to the regression of fibrosis via the release of ECM-degrading proteases. During liver fibrogenesis, parenchymal injury and the resulting inflammatory reaction generate a large panel of signals that induce the release of specific transcription factors and morphogens by quiescent HSCs; this release activates the cells and gives them fibrogenic and proinflammatory properties [3]. Thus, the HSCs’ exposure to multiple insults and/or inflammatory cytokines (such as platelet-derived growth factor (PDGF), transforming growth factor (TGF)-β, tumor necrosis factor (TNF)-α, and interleukin (IL)-1) prompts a transition from a quiescent state to an activated state.

HSC activation is a prominent determinant of hepatic immunoregulation during injury. In liver fibrosis, HSCs are important sources of TGF-β—the key paracrine or autocrine mediator responsible for greater deposition of ECM proteins [4]. It has also been reported that activated human HSCs and myofibroblasts can produce IL-6, IL-1α, IL-1β and IL-8 [5]. Furthermore, activated HSCs themselves may also produce inflammatory mediators (including chemokines) under baseline conditions or in response to signals such as TNF-α, IL-1β or lipopolysaccharide [6,7]. There is some evidence that certain chemokines (such as the CC chemokines RANTES, chemokine monocyte chemoattractant protein-1 (MCP-1/CCL2) and CCL21) directly target HSCs and thus promote cell proliferation and migration [8]. Furthermore, the recent identification of receptors for profibrogenic chemokines (including CXCR4 [9], CCR1, CCR5 [10], CXCR2 [11] and CCR2 [12]) on the surface of HSCs has enlarged the repertoire of signals promoting cell activation. The ability to block chemokine receptors with small molecule inhibitors makes HSCs ideal targets for antifibrotic therapies and reinforces the need for human-cell-based models of inflammatory signaling and inflammatory control by drugs [13].

The LX-2 cell line (developed in S. Friedman’s laboratory at the Mount Sinai School of Medicine, New York, NY) may constitute a good model of human HSCs and can thus avoid the need to use human primary cells [14]. The cell line was generated by the spontaneous immortalization of human primary HSCs (taken from a healthy donor) by low-serum incubation. LX-2 cells express α-Smooth Muscle Actin (SMA), vimentin, the intermediate filament protein glial fibrillary acidic protein, and the type β receptor for platelet-derived growth factor—suggesting that the LX-2 cells retain key features of activated/transdifferentiated HSCs. LX-2 cells also secrete pro-collagen, pro-matrix metalloproteinase (MMP)2, MT1-MMP (MMP14), Tissue Inhibitor of MetalloProteinases (TIMP)-1 and TIMP-2, all of which are characteristic features of activated HSCs [14]. In pharmacological studies, LX-2 cells have shown much the same physiological response as primary HSCs [15].

A better understanding of how the complex, inflammation-driven microenvironment can directly affect activated HSCs might lead to new antifibrotic strategies. To this end, we sought to characterize the response of LX-2 cells to activation with several pro-inflammatory cytokines (relative to activation with the well-known pro-fibrogenic mediator, TGF-β1). We focused our analysis on collagen expression, markers of fibrosis, and the MMP/TIMP imbalance. Furthermore, we evaluated the antifibrotic potential of two drugs commonly used to treat chronic inflammatory diseases (the anti-TNF-α monoclonal antibody infliximab and the IL-1 receptor (IL-1r) antagonist anakinra).

## Materials and Methods

### Reagents

Recombinant human (rh) IL-6, IL-1α, TGF-β1, TNF-α, IL-8 and granulocyte macrophage colony-stimulating factor were purchased from R&D Systems (Abingdon, UK). rhIL-1β and gelatin solution were obtained from Sigma-Aldrich (St. Louis, MO, USA). Dulbecco's Modified Eagle Medium cell culture media, antibiotics, L-glutamine, sodium pyruvate and trypsin-EDTA were purchased from Invitrogen (Eugene, OR, USA). Fetal calf serum (FCS) was from Lonza (Levallois, France). Acrylamide, sodium dodecyl sulfate (SDS), Tris, and bovine serum albumin were purchased from Eurobio (Les Ulis, France). Anti-collagen type I antibody was purchased from Millipore (Billerica, MA, USA). Anti-STAT3 and p-STAT3 antibodies were obtained from Cell Signaling Technology (Danvers, MA, USA). Anti-IL6R antibody was purchased from Abcam (Cambridge, UK). Anti-HSC70 antibody was obtained from Santa Cruz Biotechnology (Dallas, TX, USA). Polyclonal secondary immunoglobulins/HRP were purchased from Dako (Les Ulis, France). The Pierce^™^ BCA protein assay kit, LDS sample buffer, MOPS SDS running buffer, 4–12% Bis-Tris Gel, Pierce^®^ ECL western blotting substrate, antioxidant and sample reducing agent were purchased from Thermo Fisher Scientific (Saint-Herblain, France). Tris/glycine migration buffer and Trans-blot^®^ Turbo^™^ transfer pack were purchased from Bio-Rad (Hercules, CA, USA).

### Cell culture and treatments

LX-2 cells were provided by S.L. Friedman (Mount Sinai School of Medicine, New York, NY) [14]. LX-2 cells were maintained in DMEM medium supplemented with 10% FCS, 50 IU/mL penicillin, 50 μg/mL streptomycin, 2 mM L-glutamine and 1 mM sodium pyruvate at 37°C, in a humidified 5% CO_2_ incubator. The cells were used in experiments from passage 16 to passage 26.

Experiments were performed in 6-well plates. LX-2 cells were seeded at 3.10^5^ cells per well, grown in 2 mL DMEM supplemented with 10% FCS for 24 hours, and then placed in DMEM supplemented with 2% FCS for 24 hours prior to experiments. Cells were treated with rh cytokines for 24 or 48 hours.

### Extraction of total RNA and real-time PCR

Total RNA was isolated from cells using the SV Total RNA Isolation System (Promega, Madison, WI, USA). RNA quantity and purity were assessed with a Nanodrop ND-1000 spectrophotometer (Nyxor Biotech, Paris, France). Total RNA (1 μg) was reverse-transcribed into cDNA using the High-Capacity cDNA kit (Applied Biosystems, Foster City, CA, USA). Real-time quantitative RT-PCR was performed with the fluorescent dye SYBR Green methodology using the SYBR Green PCR Master Mix (Applied Biosystems) and the 7900HT fast real-time PCR system (Applied Biosystems). Primer pairs for each transcript were chosen with IDT software http://eu.idtdna.com/scitools/Applications/RealTimePCR/ and “blasted” with NCBI http://www.ncbi.nlm.nih.gov/BLAST/. Amplification curves were read with SDS 2.3 software (Applied Biosystems) using the comparative cycle threshold method. The steady-state mRNA level for each gene of interest was normalized against GAPDH mRNA.

### ELISA and gelatinase (MMP-2 and MMP-9) assays

Conditioned media were collected and stored at -20°C until use. The production of pro-collagen I α1, IL-1β, IL-8, TNF-α, and IL-6 were measured in the supernatant using a Duoset^®^ ELISA kit (R&D Systems), according to the manufacturer’s procedure. Briefly, conditioned media were added to plates coated with the appropriate capture antibody and incubated at room temperature for 2 hours, followed by incubation with the appropriate biotinylated detection antibody. Streptavidin-conjugated horseradish-peroxidase was added to the plates, and the enzyme activity after the addition of substrate was detected with an ELISA microplate reader (POLARstar Omega, BMG LABTECH, Champigny sur Marne, France). The ELISA’s sensitivity for human pro-collagen I α1, human IL-1β, human IL-8, human TNF-α, and human IL-6 were respectively 31.25–2000 pg/mL, 3.91–250 pg/mL, 31.25–2000 pg/mL, 15.63–1000 pg/mL, and 9.38–600 pg/mL.

The enzymatic activity of MMP-2 and MMP-9 into the supernatant was determined by SDS-PAGE gelatin zymography. Gelatinases present in the supernatant degrade the gelatin matrix, leaving a clear band after staining the gel for proteins. Briefly, 15 μL of supernatant were denatured in the absence of a reducing agent and electrophoresed in 7.5% SDS-PAGE containing 0.1% gelatin. The gels were incubated twice in the presence of 2.5% Triton X-100 at room temperature for 10 min, washed twice in distilled water for 20 min, and then incubated at 37°C overnight in a buffer containing 6.62 mM CaCl_2_, 2.1 μM ZnCl_2_ and 50 mM Tris (pH 8). Thereafter, the gels were stained with 0.1% Coomassie Blue, and proteolysis was detected as a white band against a blue background. The activity of MMP-2 and MMP-9 was determined by densitometric scanning of the bands and analysis using ImageJ software (Wayne Rasband, National Institute of Mental Health, Bethesda, Maryland, USA; http://rsb.info.nih.gov/ij/).

### Western blotting

LX-2 cells were treated with 3 ng/mL of rhTGF-β1for 24 h. HepaRG cells and LX-2 cells were treated with 3 ng/mL of rhIL-6 for 30 minutes or 24 h. The cells were washed with cold PBS, and finally resuspended in cell lysis buffer RIPA (50 mM Tris-HCl pH 8, 150 mM NaCl, 0,25% Sodium deoxycholate, 1 mM EDTA, 1% NP-40) supplemented with a protease inhibitor cocktail and a phosphatase inhibitor cocktail (Roche, Mannheim, Germany) and sonicated for 30 seconds. Aliquots containing equivalent total protein content, as determined by the BCA procedure with bovine serum albumin as the standard, and supplemented with reducing agent and LDS sample buffer, were subjected to SDS/4-12% polyacrylamide gel electrophoresis in MOPS SDS running buffer with antioxidant, electrotransferred to 0.2 μm nitrocellulose membranes, and incubated overnight at 4°C with primary antibodies directed against Collagen Type I, p-STAT3, STAT3, IL6R, and HSC70. After using the appropriate horseradish peroxidase conjugated antibody, membranes were incubated with Pierce^®^ ECL western blotting substrate and bands were visualized and quantified by densitometry with Fusion-Capt software (Vilber Lourmat, Fusion FX7, France).

### Statistical analysis

The results are expressed as the mean ± standard error of the mean (SEM). Intergroup differences in treatment effects were probed with an unpaired t test (for pairwise comparisons) or a one-way analysis of variance with Tukey’s *post-hoc* test (for the comparison of more than two groups). All analyses were carried out using Prism software (version 5.0, Graphpad Software, La Jolla, CA, USA). In all analyses, a two-sided *p*-value <0.05 was considered to be statistically significant.

## Results

### LX-2 cells exhibit the same expression profile as normal HSCs

**Fig 1:**
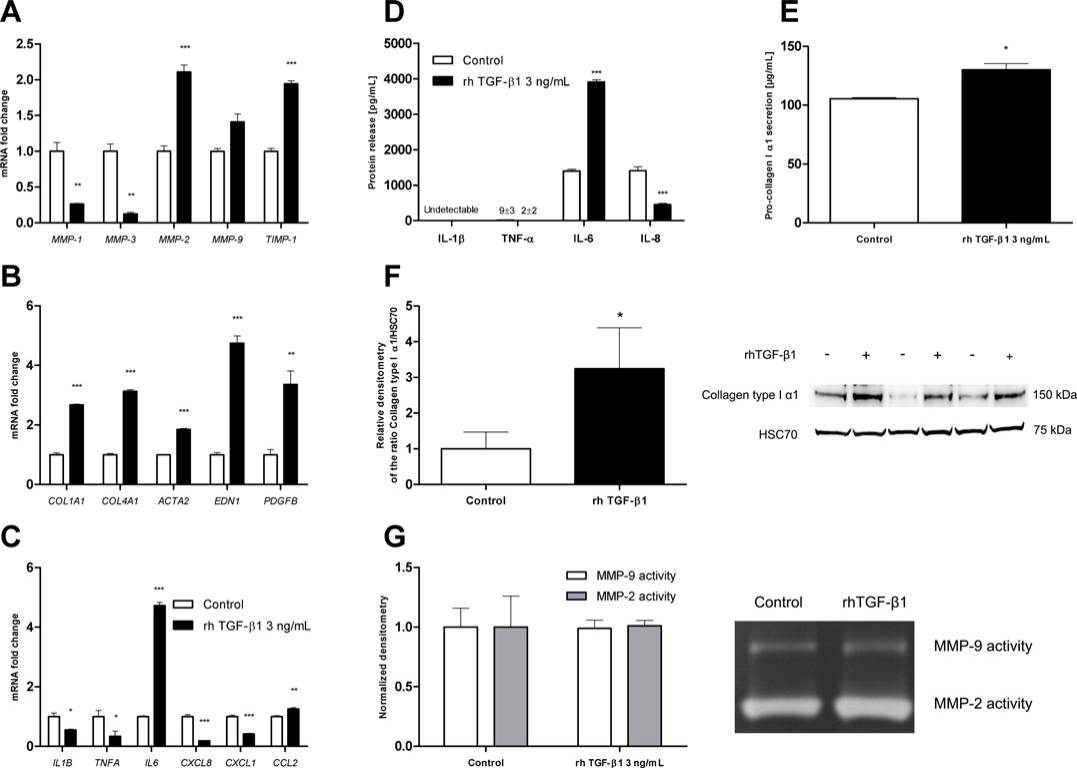
Effects of TGF-β1 treatment on the fibrolysis balance, fibrotic response and inflammatory response in LX-2 cells. The cells were treated with 3 ng/mL rhTGF-β1 for 24 hours or 48 hours. **(A)** mRNA expression levels of metalloproteinases (MMP-1, MMP-3, MMP-2, MMP-9) and tissue inhibitor of metalloproteinases (TIMP-1) were measured and normalized against that of GAPDH (using real-time PCR) after 24 hours of treatment. **(B)** mRNA expression of fibrogenesis factors (COL1A1/collagen I α1, COL4A1/collagen IV α1), myofibroblast differentiation factors (ACTA2/α-smooth muscle actin) and myofibroblast activation factors (EDN1/endothelin-1, PDGFB/platelet-derived growth factor-BB), as measured with real-time PCR and normalized against that of GAPDH after 24 hours of treatment. **(C)** mRNA expression of proinflammatory cytokines (IL1B/interleukin-1β, TNFA/tumor necrosis factor-α, IL6/interleukin-6) and chemokines (CXCL8/interleukin-8, CXCL1/GROα, CCL2/MCP1), as measured with real-time PCR and normalized against that of GAPDH after 24 hours of treatment. **(D)** IL-1β, TNF-α, IL-6 and IL-8 secretions into the supernatant by LX-2 cells were detected with an ELISA after 24 hours of treatment. **(E)** Pro-collagen I α1 secretion into the supernatant by LX-2 cells was detected with an ELISA after 48 hours of treatment. **(F)** Collagen type I α1 protein expression was determined by western blot of LX-2 cell lysates after 24 hours treatment and relative quantification was evaluated using densitometry. **(G)** Gelatinase activities of MMP-9 and MMP-2 released by LX-2 cells into the supernatant after 48 hours of treatment were evaluated by zymography and normalized densitometry. Results are expressed as the mean ± SEM of three independent experiments. * p<0.05, ** p<0.01, *** p<0.001, relative to a control.

Treatment with 3 ng/mL TGF-β1 downregulated the mRNA expression of collagenases (MMP-1 and MMP-3) by LX-2 cells, whereas the mRNA expression of gelatinases MMPs (MMP-2 and MMP-9) and TIMP-1 (the main MMP inhibitor) was upregulated (Fig 1A). LX-2 cells also expressed higher mRNA levels of myofibroblast markers (such as collagen type I α1, collagen type IV α1, α-SMA, endothelin-1 and PDGF homodimer (PDGF-BB) (Fig 1B)). Treatment with rhTGF-β1 was associated with lower mRNA levels of pro-inflammatory cytokines (IL-1β, TNF-α and IL-8) and the neutrophil chemoattractant CXCL1, and higher mRNA levels of IL-6 and the monocyte chemoattractant CCL2 (Fig 1C). These results were confirmed at protein levels by ELISA in which IL-8 release was downregulated and IL-6 release upregulated after 24 hours treatment with rhTGF-β1. However, IL-1β levels were not detectable and TNF-α levels were barely detectable in both treated and control conditions (Fig 1D). The ELISA results showed that LX-2 cells were able to release large amounts of pro-collagen type I α1 into the supernatant. This release was enhanced by treatment with rhTGF-β1 for 48 hours (Fig 1E), correlated with the upregulation of protein expression of collagen I as observed with western blot (Fig 1F). Furthermore, the zymography results showed that gelatinase (MMP-2 and MMP-9) activity was not enhanced (Fig 1G).

### IL-6 has no effect on LX-2 cells

**Fig 2:**
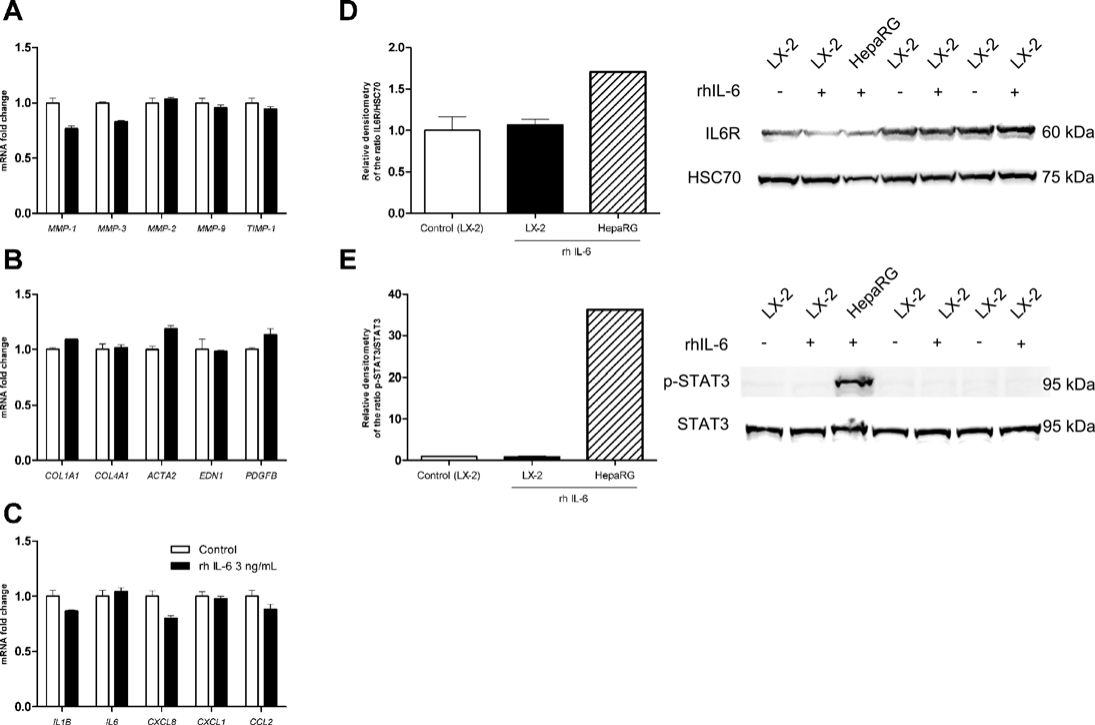
Effects of IL-6 treatment on the fibrolysis balance, fibrotic response and inflammatory response in LX-2 cells. The cells were treated with at 3 ng/mL rhIL-6 for 30 minutes or 24 hours. **(A)** mRNA expression of metalloproteinases (MMP-1, MMP-3, MMP-2, MMP-9) and tissue inhibitor of metalloproteinases (TIMP-1), as measured with real-time PCR and normalized against that of GAPDH after 24 hours of treatment. **(B)** mRNA expression of fibrogenesis factors (COL1A1/collagen I α1, COL4A1/collagen IV α1), myofibroblast differentiation factors (ACTA2/α-smooth muscle actin) and myofibroblast activation factors (EDN1/endothelin-1, PDGFB/platelet-derived growth factor-BB), as measured with real-time PCR and normalized against that of GAPDH after 24 hours of treatment. **(C)** mRNA expression of proinflammatory cytokines (IL1B/interleukin-1β, IL6/interleukin-6) and chemokines (CXCL8/interleukin-8, CXCL1/GROα, CCL2/MCP1), as measured with real-time PCR and normalized against that of GAPDH after 24 hours of treatment. **(D)** IL6R protein expression was determined by western blot on HepaRG and LX-2 cells lysates after 24 hours treatment and relative quantification was evaluated using densitometry. **(E)** p-STAT3 protein expression was determined by western blot on HepaRG (positive control) and LX-2 cells lysates after 24 hours treatment and relative quantification was evaluated using densitometry. Results are expressed as the mean ± SEM of three independent experiments.

Treatment of LX-2 cells with 3 ng/mL of IL-6 for 24 hours was not associated with high or lower mRNA expression levels (relative to control experiments) of MMP-1, MMP-3, MMP-2, MMP-9, TIMP-1 (Fig 2A), collagen types I and IV α1, endothelin-1, PDGF-BB, α-SMA (Fig 2B), IL-1β, IL-6, IL-8, CXCL1 or CCL2 (Fig 2C). The absence of the IL6 receptor in LX-2 could be an explanation for these results. However, we showed that IL6R was detectable by western blot in LX-2 cells at a level comparable to that in HepaRG cells (Fig 2D). IL-6 treatment did not induce the upregulation of this receptor in LX-2 cells (Fig 2D), and also did not induce the phosphorylation of STAT3 in LX-2 cells while it was observed in HepaRG cells (Fig 2E).

### CXCL8/IL-8 and TGF-β1 have opposite effects on LX-2 cells

**Fig 3:**
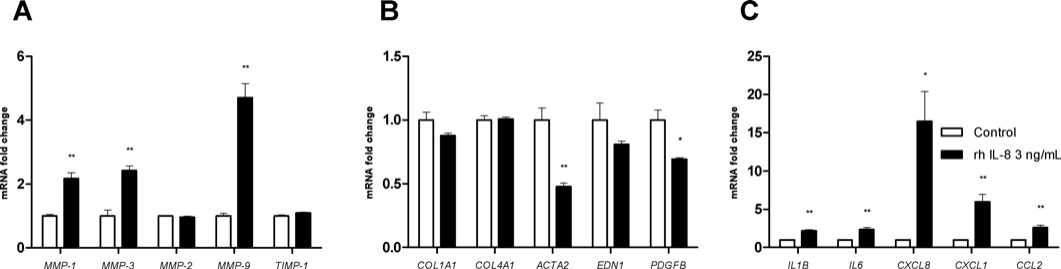
Effects of CXCL8/IL-8 treatment on the fibrolysis balance, fibrotic response and inflammatory response in LX-2 cells. The cells were treated with 3 ng/mL rhIL-8 for 24 hours or 48 hours. **(A)** mRNA expression of metalloproteinases (MMP-1, MMP-3, MMP-2, MMP-9) and tissue inhibitor of metalloproteinases (TIMP-1), as measured with real-time PCR and normalized against that of GAPDH after 24 hours of treatment. **(B)** mRNA expression of fibrogenesis factors (COL1A1/collagen I α1, COL4A1/collagen IV α1), myofibroblast differentiation factors (ACTA2/α-smooth muscle actin) and myofibroblast activation factors (EDN1/endothelin-1, PDGFB/platelet-derived growth factor-BB), as measured with real-time PCR and normalized against that of GAPDH after 24 hours of treatment. **(C)** mRNA expression of proinflammatory cytokines (IL1B/interleukin-1β, IL6/interleukin-6) and chemokines (CXCL8/interleukin-8, CXCL1/GROα, CCL2/MCP1), as measured with real-time PCR and normalized against that of GAPDH after 24 hours of treatment. Results are expressed as the mean ± SEM of three independent experiments. * p<0.05, ** p<0.01, relative to a control.

Treatment with 3 ng/mL IL-8 was associated with mRNA upregulation of MMP-1, MMP-3 and MMP-9 but not MMP-2 and TIMP-1 (Fig 3A). IL-8 did not induce a relative reduction of mRNA expression of the collagen types I and IV α1 or endothelin-1 but was associated with lower mRNA levels for α-SMA and PDGF-BB (Fig 3B). Lastly, we observed that IL-8 was associated with greater mRNA expression of IL-1β, IL-6, IL-8, CXCL1 and CCL2 in LX-2 cells (Fig 3C).

### TNF-α and CXCL8/IL-8 have similar effects on LX-2 cells

**Fig 4:**
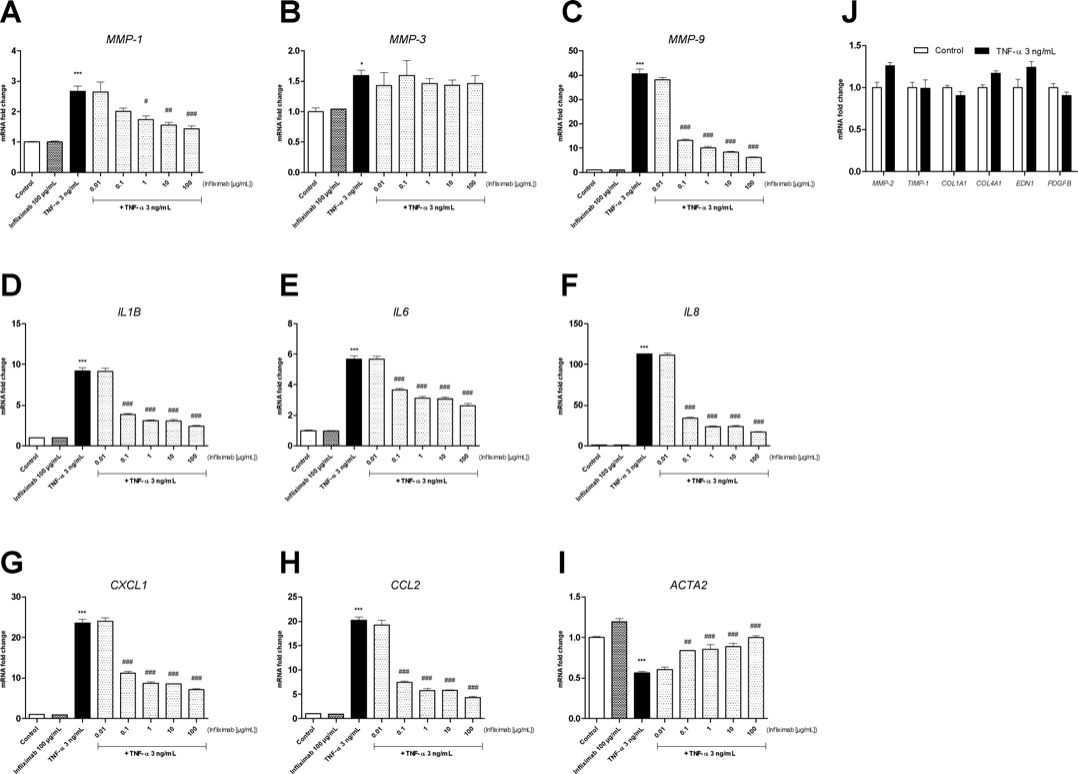
Effects of TNF-α treatment on the fibrolysis balance, fibrotic response and inflammatory response in LX-2 cells. The cells were pre-treated (or not) with the anti-TNF-α monoclonal antibody infliximab (at a dose ranging from 0.01 to 100 μg/mL) and then treated with 3 ng/mL rhTNF-α for 24 hours. **(A)** mRNA expression of metalloproteinases (MMP-1, MMP-3 and MMP-9) myofibroblast differentiation factors (ACTA2/α-smooth muscle actin), and proinflammatory cytokines (IL1B/interleukin-1β, IL6/interleukin-6) and chemokines (CXCL8/interleukin-8, CXCL1/GROα, CCL2/MCP1), as measured with real-time PCR and normalized against that of GAPDH after 24 hours of treatment. **(B)** mRNA expression of the tissue inhibitor of metalloproteinases (TIMP-1), MMP-2, fibrogenesis factors (COL1A1/collagen I α1, COL4A1/collagen IV α1) and myofibroblast activation factors (EDN1/endothelin-1, PDGFB/platelet-derived growth factor-BB), as measured with real-time PCR and normalized against that of GAPDH after 24 hours of treatment. Results are expressed as the mean ± SEM of three independent experiments. * p<0.05, *** p<0.001, relative to a control. # p<0.05, ## p<0.01, ### p<0.001, relative to TNF-α.

Treatment with 3 ng/mL TNF-α was associated with upregulation of MMP-1, MMP-3, MMP-9, IL-1β, IL-6, IL-8, CXCL1 and CCL2 mRNA expression, and downregulation of α-SMA mRNA expression (Fig 4A–4I). There were no differences (relative to control experiments) in mRNA expression levels of MMP-2, TIMP-1, collagen types I and IV α1, endothelin-1 and PDGF-BB (Fig 4J). Furthermore, we observed that infliximab (a monoclonal antibody that targets soluble or membrane-bound forms of human TNF) inhibited the effects of TNF-α on LX-2 cells at a concentration of 1 µg/mL (for *MMP-1*) and 0.1 µg/mL (for *MMP-9*, *IL1B*, *IL6*, *IL8*, *CXCL1*, *CCL2*, and *ACTA2)*. However, infliximab treatment did not inhibit the expression of *MMP-3*; this suggests that TNF-α induces the production of another factor that in turn regulates the expression of MMP-3 at the mRNA level (Fig 4B).

### IL-1β and IL-1α have similar effects on LX-2 cells

**Fig 5:**
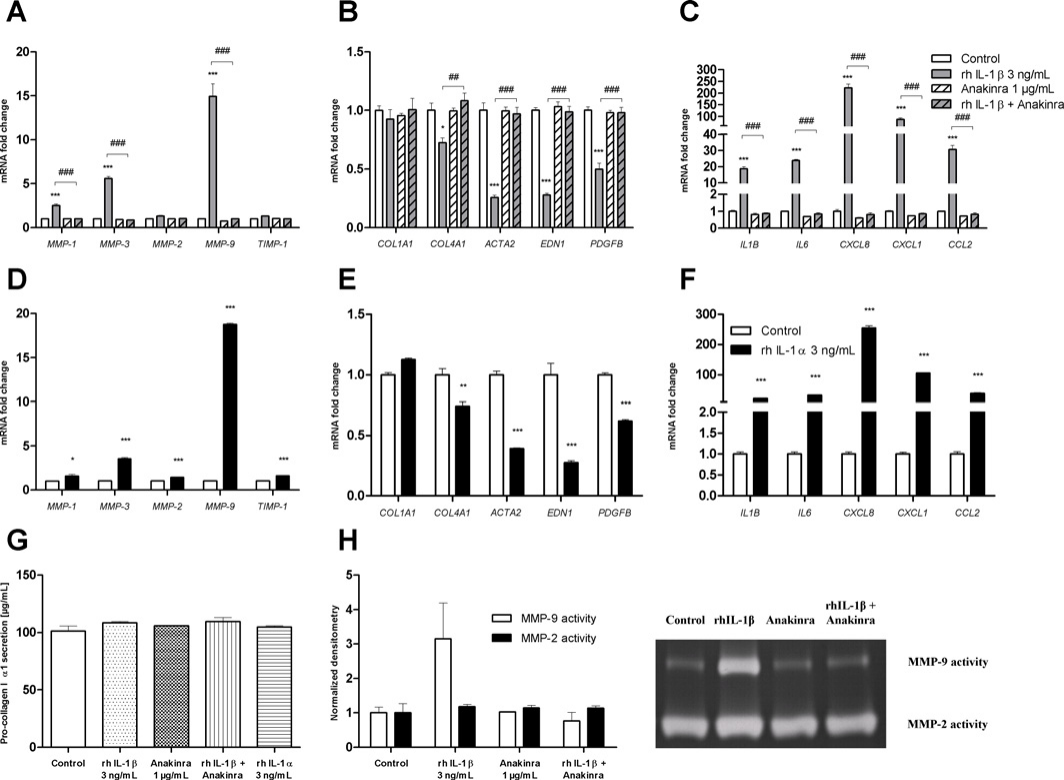
Effects of IL-1β and IL-1α treatment on the fibrolysis balance, fibrotic response and inflammatory response in LX-2 cells. The cells were pre-treated (or not) with the IL-1 receptor antagonist anakinra (1 μg/mL) and then treated with 3 ng/mL rhIL-1β or 3 ng/mL rhIL-1α for 24 hours or 48 hours. **(A, D)** mRNA expression of metalloproteinases (MMP-1, MMP-3, MMP-2, MMP-9) and tissue inhibitor of metalloproteinases (TIMP-1), as measured with real-time PCR and normalized against that of GAPDH after 24 hours of treatment. **(B, E)** mRNA expression of fibrogenesis factors (COL1A1/collagen I α1, COL4A1/collagen IV α1), myofibroblast differentiation factors (ACTA2/α-smooth muscle actin) and myofibroblast activation factors (EDN1/endothelin-1, PDGFB/platelet-derived growth factor-BB), as measured with real-time PCR and normalized against that of GAPDH after 24 hours of treatment. **(C, F)** mRNA expression of proinflammatory cytokines (IL1B/interleukin-1β, IL6/interleukin-6) and chemokines (CXCL8/interleukin-8, CXCL1/GROα, CCL2/MCP1), as measured with real-time PCR and normalized against that of GAPDH after 24 hours of treatment. **(G)** Pro-collagen I α1 secretion into the supernatant by LX-2 cells was detected with an ELISA after 48 hours of treatment. **(H)** Gelatinase activities of MMP-9 and MMP-2 released by LX-2 cells into the supernatant after 48 hours of treatment were evaluated by zymography and normalized densitometry. The results are expressed as the mean ± SEM of three independent experiments. * p<0.05, ** p<0.01, *** p<0.001, relative to a control. # p<0.05, ### p<0.001, relative to IL-1β.

IL-1β (3 ng/mL) upregulated mRNA expression for MMP-1, MMP-3 and MMP-9 but not for MMP-2 and TIMP-1 (Fig 5A). It downregulated mRNA expression of collagen type IV α1, α-SMA, endothelin-1 and PDGF-BB but did not modify collagen type I α1 expression (Fig 5B). Lastly, we observed that IL-1β upregulated mRNA expression of itself, IL-6, IL-8, CXCL1 and CCL2 in LX-2 cells (Fig 5C). All the effects of rhIL-1β were totally inhibited by a 1-hour pretreatment of LX-2 cells with 1 μg/mL of the IL-1 receptor antagonist anakinra. rhIL-1α has the same affinity as rhIL-1β for the IL-1 receptor and was used at a concentration of 3 ng/mL to treat LX-2 cells. rhIL-1α was associated with same changes as IL-1β in terms of the mRNA expression of MMPs and TIMPs (except for MMP-2 and TIMP-1, which were upregulated) (Fig 5D), fibrogenic markers (Fig 5E) and inflammatory cytokines and chemokines (Fig 5F). We confirmed that neither IL-1β nor IL-1α treatment (for 48 hours) was associated with the greater release of pro-collagen type I α1 into the supernatant (Fig 5G).

## Discussion

The present study investigated LX-2 cells’ expression of the chemokines IL-8, CCL2/MCP-1 and CXCL1/GROα and the cytokines IL-1β and IL-6 in response to treatment with pro-inflammatory cytokines and chemokines (such as IL-1β, IL-1α, IL-8 and TNF-α). We found that the LX-2 HSC cell line became pro-fibrogenic when treated with TGF-β1 and anti-fibrogenic when treated with IL-1β. Indeed, the pro-fibrogenic vs. anti-fibrogenic environment in LX-2 cells appeared to depend on the MMP/TIMP imbalance and the latter’s impact on collagen production. Moreover, experiments with anakinra and infliximab showed that the pro-inflammatory cytokines IL-1β and TNF-α are major players in fibroblast/macrophage mediated chronic liver fibrosis. These drugs may therefore be of value for the treatment of liver diseases.

Activated HSCs contribute significantly to liver fibrogenesis through proliferation, chemotaxis, ECM synthesis and contractility. In recent years, it has become clear that HSCs are prominent determinants of hepatic immunoregulation during injury. The cells express a battery of chemokines (including CCL2, CCL5, CXCL2, CXCL8, CXCL9, CXCL10, CXCL12, and CX3CL3) known to recruit neutrophils, macrophages/monocytes, natural killer cells, dendritic cells, natural killer T cells and other T cells [16]. Furthermore, activated HSCs secrete inflammatory mediators in response to signals such as TNF-a, IL-1beta, and lipopolysaccharide [6,7]. Hence, HSCs amplify the inflammatory response in a context of liver disease.

TGF-β is a well-characterized, pro-fibrotic cytokine that activates HSCs, induces the latter’s expression of matrix-producing genes and inhibits degradation of ECM by downregulating MMP expression and promoting TIMP expression; this leads to the excessive deposition of collagen fibers and promotes liver fibrosis [17,18]. In line with the literature data, our present results showed that HSCs LX-2 cells are able to respond to TGF-β1 by increasing the mRNA expression of MMP-2, TIMP-1, α-SMA, endothelin-1, PDGF-BB, type IV collagen α1 and type I collagen α1 at the mRNA and protein levels. TGF-β1 also downregulated mRNA and protein levels of MMP-1, MMP-3, IL-1β, TNF-α, CXCL1 and CCL2. Surprisingly, we found that TGF-β1 was able to induce IL-6 expression. In view of the properties of TGF-β (which is both fibrogenic and immunosuppressant), potent pro-inflammatory cytokines like IL-1β or TNF-α are unlikely to display pro-fibrogenic activity.

We also observed that the treatment of LX-2 cells with TNF-α upregulated the mRNA expression of inflammatory cytokines and chemokines, MMP-1, MMP-3 and MMP-9 and downregulated mRNA expression of α-SMA. This is consistent with a report of TNF-α’s direct antifibrotic effect on HSCs [19]. Our results also showed that TNF-α stimulates the release of MMPs and thereby prevents the accumulation of ECM. However, we did not observe low mRNA expression of fibrotic marker genes (pro-collagen type I α1, MMP-2, TIMP-1, endothelin-1 and PDGF-BB and pro-collagen IV α1). We also found that IL-1β and IL-1α have the same effects as TNF-α on LX-2 cells. Moreover, IL-1 signaling had an antifibrogenic effect on LX-2 cells, with upregulation of MMP expression and MMP-9 activity, no difference in MMP-2 and TIMP-1 expression levels, and downregulation of pro-collagen IV α1, α-SMA, endothelin-1 and PDGF-BB. However, we cannot conclude that IL-1β and IL-1α have antifibrogenic role because treatments with these cytokines were not associated with differences in mRNA or protein levels of pro-collagen I α1. However, it has been shown that MMP-9 induces the maturation of TGF-β [20], thereby improving fibrogenesis in the longer term.

IL-6 is a pleiotropic cytokine involved in inflammation, hematopoiesis and immune regulation. Treatment with IL-6 reportedly reduces carbon tetrachloride (CCl_4_)-induced acute and chronic liver injury and fibrosis [21]. Furthermore, elevated blood levels of IL-6 have been observed in patients with NAFLD, and it is thought that IL-6 can induce insulin resistance and inflammation in the liver [22,23]. These observations indicated that IL-6 has a role in the development of NAFLD. Nevertheless, IL-6 did not have any effect on LX-2 cells in the present study—suggesting that this cytokine is not a valuable factor in the treatment of chronic liver disease. However, we showed that LX-2 cells express the IL6 receptor, but failed to induce the phosphorylation of the downstream signaling STAT3 pathway. These results suggested that this receptor is ineffective, which might be due to an impairment of the glycoprotein 130. Indeed, this protein is essential for the signal transduction following cytokine engagement.

The chemokine IL-8 is produced by a variety of neutrophil-activating cells. Peripheral neutrophilia and liver neutrophil infiltration are frequently noted in patients with alcoholic liver disease [24,25]. Individuals with advanced liver disease express abnormally high levels of IL-8 [26,27], and elevated serum levels of IL-8 are correlated with a higher mortality rate. Our present results show that like TNF-α and IL-1, IL-8 have some antifibrogenic effects opposed to TGF-β1, in which it upregulated the mRNA expression levels of various cytokines/chemokines and MMP-1, MMP-3 and MMP-9 in LX-2 cells. In contrast, IL-8 treatment was associated with the downregulation of α-SMA and PDGF-BB mRNA expression—suggesting a more complex role for this chemokine in liver disease.

We also evaluated the potential anti-inflammatory effects of infliximab on the stimulation of LX-2 cells by TNF-α. Indeed, circulating levels of TNF-α are elevated in patients with liver fibrosis and are associated with a poor prognosis [28]. Experiments in a variety of models and diseases have shown that TNF-α accentuates liver fibrosis by increasing hepatocellular damage. TNF-α mediates alcohol- or dimethylnitrosamine-induced liver injury in animal models [29,30] and has an important role in the perpetuation of HSC activation *in vitro* and the synthesis of ECM [31–33]. Furthermore, the TNF-α-induced liver failure and exacerbation of liver damage following exposure to the hepatotoxin CCl_4_ were abrogated by treatment with a soluble TNF receptor [34] and were inhibited in TNF-knockout mice [35]. TNF-α is also known to induce fibrosis in other models. In a model of pulmonary fibrosis, TNF-receptor-knockout mice were protected from the development of fibroproliferative lesions [36]. Reducing TNF-α production or blocking TNF-α’s action significantly minimizes liver injury caused by alcohol toxicity, acetaminophen overdose or ischemia/reperfusion-associated liver injury in animal models [37–40]. Infliximab has been tested in the treatment of severe alcoholic hepatitis [41]; although it reduced liver damage and neutrophil infiltration, the possible attenuation of fibrosis was not evaluated. We presently report that TNF-α treatment of LX-2 cells was associated with differences (relative to control experiments) in the mRNA expression of genes coding for inflammatory cytokines/chemokines, MMPs and α-SMA. Furthermore, treatment with infliximab was associated with lower expression of most of the studied genes (with the notable exception of MMP-3). However, cytokine and chemokine expression levels remained high.

With regard to the observed effects of IL-1, we also analyzed the effect of anakinra. In fact, IL-1β has recently come back under the spotlight in the field of liver disease. IL-1 can directly activate HSCs and stimulate them to produce MMP-9, MMP-13 and TIMP-1, resulting in liver fibrogenesis. In contrast, IL-1R-knock-out mice are less likely to sustain liver damage or develop fibrosis [42]. Furthermore, the machinery of the inflammasome pathway (which regulates the maturation and liberation of IL-1β) is known to be expressed by HSCs, and mice lacking inflammasome components suffer from lower levels of CCl_4_- and TAA-induced liver fibrosis [43]. IL-1α or IL-1β knock-out mice are also less likely to develop liver fibrosis in animal models of steatohepatitis [44]. Petrasek and colleagues have shown that blockade of the IL-1 receptor 1 in a rodent model of alcoholic liver disease significantly reduced steatosis, inflammation and injury [45]. Similarly, IL-1 receptor antagonists were found to protect rats against the development of dimethylnitrosamine-induced liver fibrosis [46], and blocking IL-1 signaling was able to markedly attenuate alcohol-induced liver inflammation and pro-steatotic MCP-1/CCL2 levels in hepatocytes, and increases Toll-like-receptor-4-dependent upregulation of inflammatory signaling in macrophages [45].

We also found that IL-1β and IL-1α had the same effects on LX-2 cells, which are similar to the effects of TNF-α. The fact that the IL-1 receptor antagonist anakinra markedly inhibited IL-1β’s effects on LX-2 cells suggests that this drug could be tested as an anti-inflammatory in patients with liver disease. In contrast to anti-TNF approaches (the value of which is compromised by infectious complications) [47,48], anakinra has an excellent safety profile. Its use has not been associated with adverse reactions or superinfections during the long-term treatment of patients with rheumatoid arthritis [49] or in the acute treatment of patients with sepsis [50]. Further research in the field of liver disease is therefore justified. Much as with anakinra, we also found that the anti-TNF-α agent infliximab was associated with differences in the mRNA expression of genes coding for inflammatory cytokines/chemokines, MMPs and α-SMA. Infliximab downregulated the expression of genes for cytokines, chemokines and various MMPs. This finding also suggests that infliximab should be tested as a treatment for chronic liver diseases. However, the LX-2 cells’ expression levels of cytokines and chemokines were significantly higher after anakinra treatment than after infliximab treatment.

In conclusion, our present results clearly show that LX-2 HSCs become pro-fibrogenic after treatment with TGF-β1 and anti-fibrogenic after treatment with IL-1β. These pro- and anti-fibrogenic effects were clearly dependent on the LX-2 cells’ MMP/TIMP imbalance and production of collagen. Moreover, experiments with anakinra and infliximab showed that the pro-inflammatory cytokines IL-1β and TNF-α are significantly involved in the pro-inflammatory process that controls fibroblast/macrophage-mediated chronic liver fibrosis. These findings also suggest that infliximab and (to a lesser extent) anakinra may be of value as treatment options in patients with chronic liver disease.

## Supporting Information

S1 Table Primers used in this study for real-time PCR assay. (DOCX)
