# Supplementary material for: Characterization of the MMP/TIMP Imbalance and Collagen Production Induced by IL-1β or TNF-α Release from Human Hepatic Stellate Cells
Source: PLoS One. 2016 Apr 5;11(4):e0153118. doi: 10.1371/journal.pone.0153118 (PMC4821480; doi:10.1371/journal.pone.0153118)
Supplement: S1 Table — (DOCX) [file pone.0153118.s001.docx]

**S1 Table.** **Primers used in this study for real-time PCR assay.**

| **Human Gene name** | **Forward sequences 5’ -> 3’** | **Reverse sequences 5’ -> 3’** |
| --- | --- | --- |
| **GAPDH** | ATGACATCAAGAAGGTGGTG | CATACCAGGAAATGAGCTTG |
| **MMP1** | ATGTGGCTCAGTTTGTCCTC | GCTTTCTCAATGGCATGGTC |
| **MMP2** | AAGTATGGCTTCTGCCCTGA | ATTTGTTGCCCAGGAAAGTG |
| **MMP3** | GACAAAGGATACAACAGGGAC | TGAGTGAGTGATAGAGTGGG |
| **MMP9** | CGAACTTTGACAGCGACAAG | CACTGAGGAATGATCTAAGCCC |
| **TIMP1** | CAAGATGTATAAAGGGTTCCAAGC | TCCATCCTGCAGTTTTCCAG |
| **ACTA2** | CATCCTCATCCTCCCTTGAG | ATGAAGGATGGCTGGAACAG |
| **COL1A1** | CCGGCTCCTGCTCCTCTTAGCG | CGTTCTGTACGCAGGTGATTGGTGG |
| **COL4A1** | CCTGGCTTGAAAAACAGCTC | CCCTGCTGAGGTCTGTGAAC |
| **EDN1** | CTTCGTTTTCCTTTGGGTTCAG | GCTCAGCGCCTAAGACTG |
| **PDGFB** | ATGATCTCCAACGCCTGC | TCAGCAATGGTCAGGGAAC |
| **IL1B** | ATGCACCTGTACGATCACTGA | ACAAAGGACATGGAGAACACC |
| **TNFA** | ACTTTGGAGTGATCGGCC | GCTTGAGGGTTTGCTACAAC |
| **IL6** | AAAGAGGCACTGGCAGAAAA | TTTCACCAGGCAAGTCTCCT |
| **IL8** | AAATTTGGGGTGGAAAGGTT | AAGAAACCACCGGAAGGAAC |
| **CXCL1** | AACCGAAGTCATAGCCACAC | CCTCCCTTCTGGTCAGTTG |
| **CCL2** | TGTCCCAAAGAAGCTGTGATC | ATTCTTGGGTTGTGGAGTGAG |
